# Reflection of mentors and mentees at initiation of Faculty Mentorship Program at Aga Khan University: A perspective

**DOI:** 10.12669/pjms.38.6.5454

**Published:** 2022

**Authors:** Rehana Rehman, Fauzia Khan, Naila Kayani, Tazeen Saeed Ali

**Affiliations:** 1 Dr. Rehana Rehman, MBBS, M. Phil, Ph.D., FHEA (UK) Associate Professor, Department of Biological & Biomedical Sciences Aga Khan University, Karachi, Pakistan; 2 Dr. Naila Kayani, Prfessor of Pathology & Medicine Aga Khan University, Karachi, Pakistan; 3 Dr. Fauzia Khan, MBBS, FRCA Professor, Department of Anesthesiology Aga Khan University, Karachi, Pakistan; 4 Dr. Tazeen Saeed Ali, Professor & Assistant Dean School of Nursing & Midwifery Aga Khan University, Karachi, Pakistan

**Keywords:** Mentorship, Mentors, Mentees, Evaluation of faculty mentorship programs

## Abstract

**Objectives:**

To explore perception of mentors and mentees about ‘Mentorship Program at Aga Khan University Medical College (AKU-MC) from a structured feedback form

**Methods:**

A retrospective study was conducted for evaluation of mentorship program at AKU-MC during the period from Jan 2019 to March, 2021. Responses on validated “Pre-intervention Probe Forms”, from forty-seven mentors and fourteen mentees inducted in the program were reviewed. Confidentiality and anonymity of data were deliberated. All replies to each question were entered in a separate worksheet to determine the frequency and percentage of answers. Responses conveying same message, but worded differently were then grouped.

**Results:**

All the mentees (n=14) responded positively to the question on the “understanding of the mentoring program. The mentees (n=12, 86%) recognized the potential of the program to transfer knowledge and skills, (n=11, 79%) supported its role for achievement of goals, (n=7, 50%), acknowledged its role in faculty relationships. The mentors expressed their enthusiasm to help the mentee’s in their professional development. They (n=20, 43%) offered support to set career goals, (n=29, 62%) proposed transfer of knowledge, skills, and experiences to achieve goals, (n=15, 32%) decided to be “role models”. Some (n=10, 21%) forecasted improved communication skills, (n=14, 30%) boosted leadership capabilities, (n=13, 28%) expected improved work performance, (n=15, 32%) opinioned that networking and leadership qualities will impact the growth of the mentee to meet the university’s expectations.

**Conclusion:**

Both mentors and mentees recognized the importance of the faculty mentorship program at AKU-MC for professional guidance, development and improvement in work performance.

## INTRODUCTION

*Mentoring is: “A process whereby an experienced, highly regarded, empathetic person (the mentor) guides another (usually younger) individual (the mentee) in the development and re-examination of their ideas, learning, and personal and professional development. The mentor, who often (but not necessarily) works in the same organization or field as the mentee, achieves this by listening or talking in confidence to the mentee”*.^1^ Mentoring has progressed as an essential technique to develop and sustain faculty in academic professions.^2^ The need for faculty mentoring has been realized at every step of early, mid-career, and senior faculty development.^3^

Faculty development programs in medical education commenced formally in 1990s in the USA.^1^ Developing countries like India, Pakistan, Bangladesh, Sri Lanka, and Afghanistan have 500 medical colleges with scanty structured mentorship programs.^1^ Mentorship is crucial for career development as it improves job satisfaction and academic productivity, promotes institutional belonging, and increases faculty retention. Effective mentorship has been linked to mentee’s productivity, self-efficacy, promotion, and career satisfaction. It also manages time effectively,and promotes higher academic self–sufficiency. Inadequate and informal mentorship on the other hand is one of the important obstacles, that threatens the successful career of faculty and the progress of the institute.^4^

Informal mentoring program was already in place at Aga Khan University. Keeping in mind the importance of formal mentoring program and its role in personal and professional development of faculty, formal mentorship program at Aga Khan University Medical College (AKU-MC) was initiated in 2019. The aim was to create a conducive learning environment for faculty empowerment and development. The specific goals were to offer an opportunity for new joining faculty to be attached to senior faculty for a deeper understanding of the culture and vision of AKU, so as to translate this into work and practice. Also, aimed to learn how different departments work and the collaborative approaches that could carry AKU’s vision forward making it a “center of excellence” in different services.

### Rationale

Literature suggests that exploration of the mentorship programs is fundamental to assess the structure, processes (matching of mentors- mentees), goal settings, resources, strengths, weaknesses, and challenges faced by the programs.^5^ The valuable self-reflection from mentors and mentees can help to devise strategies for program improvements enhancing mentor-mentee growth, and reputation of the organization.

This study therefore aimed to explore the responses obtained from both mentors and mentees on their expectations from a formal mentorship program at AKU-MC.The responses obtained from mentors and mentees therefore will help in identifying issues in the relationship and measures to improve the outcome. It will enrich mentorship culture at AKU-MC which may help in commitment, contentment and retention of faculty members in the institution

## METHODS

After acquiring exemption from the Ethical Review Committee of Aga Khan University, (ERC-AKU- 2021-6156-18199 13th June 2021) we conducted a retrospective review and evaluated responses from mentors and mentees on their expectations from the formal Faculty Mentorship program during the period from January 2019 to March 2021. The instrument was a questionnaire designed separately for mentors and mentees (“Pre-intervention Probes for Mentors” and “Pre-intervention Probes for Mentees”) by the “Medical College Mentorship Forum”. It was validated by Delphi Rounds in which two experts from department of medical education and two experienced mentors were invited. The experts reviewed the tool and marked all those items which according to their opinion should not be part of the tool, those that needed editing or rephrasing were also highlighted, items which need exclusion were also marked. On the basis of their recommendations, rrepetitions were omitted, simple, clear, and unambiguous instructions and descriptors for assessors were added before administration. It was sent to both mentors and mentees who opted to join the program (from January 2019 to March 2021). The Mentee’s form comprised of five probing questions, regarding their perceptions of the mentorship program, whereas the forms for mentors had four questions.

Pre-intervention Probes for Mentees

Q1. What is your understanding of “mentoring program”?

Q2. What are your expectations from the mentor?

Q3. How frequent are you expecting to meet the mentor in a year?

Q4. What challenges do you perceive in being a mentee?

Q5. Will you prefer a mentor from outside your section or department?

Pre-intervention Probes for Mentors

Q1. What is your understanding of “mentoring program”?

Q2. What are your expectations from the mentee?

Q3. How do you think the mentoring program will help in your mentee’s professional development?

Q4. What challenges do you perceive in being a mentor?

Forty-seven mentors (associate professors and above) who had agreed to join the mentors pool, and fourteen mentees at the level of senior instructors and assistant professors enrolled in the formal AKU-MC mentorship program and agreed to fill the preintervention probes. Faculty members who did not fill the pre-intervention probe were not included. Responses were collected via emails. To maintain confidentiality, the forms were anonymized before data interpretation and the names of mentors and mentees were not shared with the principal investigator. The responses were entered in separate Excel spreadsheets. All replies to each question were entered in a separate worksheet to determine the frequency and percentage of answers. Responses conveying the same message but worded differently were then grouped.

## RESULTS

**Fig.1:**
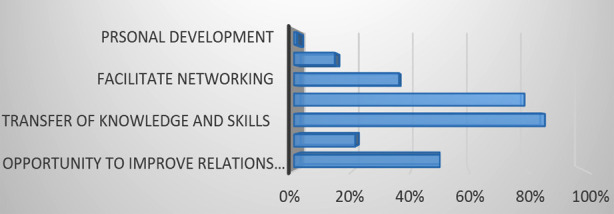
Mentees Perception of Mentorship Program.

The details of study participants is covered in Table-I Analyses of the “Mentees” perception of the mentorship program: All the mentees (n=14) responded positively to the question on the “understanding of the mentoring program”. One verbalized it as, “*The program should be smart enough to support, develop, enhance or even serve as a catalyst to achieve mentee’s professional goals*”. Another said, “*The program will offer opportunity for new joining faculty to be tagged with a senior faculty to understand the culture and vision of AKU*.” The majority (n=12) recognized the potential of the program to effectively transfer knowledge and skills, and help in the achievement of the mentee’s goals. They responded that partnership will also improve relationships between the senior and junior faculty thus facilitating networking, and acquainting the new entrants to AKUs culture and work practices (Fig.1). Regarding the “expectations from the mentors” the mentees expected assistance in both professional and personal development, however the frequency of meetings suggested per year varied.

**Table-I:** Dempgraphic Characterstics of Study Participants.

Description	Male	Female	Total	Age Range (years)	Experience at AKU Range (years)
Mentors	20	27	47	38 -- 67	2 - 27
Mentees	4	10	14	32 -- 42	1- 10

### Analyses of the “Mentors” perception of the mentorship program

**Fig.2:**
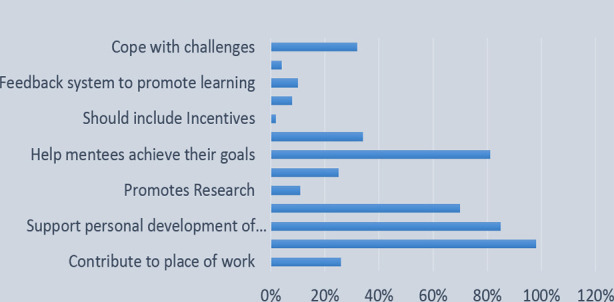
Mentors perception of the mentorship program.

Mentors (n=46) were very sure about the contribution of the mentorship program to the mentees’ professional development and (n=40) suggested both professional and personal development thus affecting overall productivity at the workplace. One stated it as *“Nurturing a young mind, facilitating them to achieve goals, providing directions and getting them to overcome hurdles, providing technical support whenever possible”*. Others (n=38) mentioned that this partnership would assist the mentees learn to set and achieve goals, facilitate networking, liaise with different departments and guide research work presentation or publications (Fig.2).

### Roles of mentoring program in mentee’s professional development

The mentors were displaying positivity and enthusiasm in their responses to this question as e.g. *“The mentor’s life experiences will be realistic aspirations for the mentee”. “The program will help junior faculty in developing a niche for themselves at AKU and have someone guide them through their choices for professional and personal development”*. They (n=20, 43%) offered support to set career goals, (n=29, 62%) proposed transfer of knowledge, skills, and experiences to achieve goals, (n=11, 23%) sponsored new ideas and thinking, (n=15, 32%) decided to be “role-models”. Some (n=14, 30%) forecasted improved communication skills, enhanced leadership capabilities, improved feedback mechanism, and predicted support in research presentations and publications. They (n=13, 28%) mentioned that networking and leadership qualities will reduce stress and improve work performance enabling the mentee to meet university’s expectations.

A lot of responses from both mentors and mentees came up as “fears” and “challenges” in following up the program. Mentees (n=8, 57%) feared time management, (n=4, 29%) worried a balance of expectations versus role, (n=3, 21%) were uneasy about performance challenges, personality mismatch, while (n=1, 7%) feared honest and trustworthy relations. On the other hand mentors (n=26, 55%) were concerned only about the mentees commitment to take a proactive role in scheduling meetings, complying with feedback and maintaining honest and trustworthy relationship.

## DISCUSSION

A formal mentoring program is not only a cost-effective solution to counterbalance the worldwide scarcity of health educators and health professionals but also provides the infrastructure to achieve specific organizational goals. Best practices in mentorship can provide the best resolution to faculty retention. The study recognized the potential of the AKU Mentorship program in knowledge and skills transfer, advocated on professional and personal development, and acknowledged its role in developing faculty relationships. Mentees perceived the role of mentors in effective teaching, and productive research^3^ and both highlighted the significance of organizational support in defining, executing, and evaluating the objectives of the mentoring relationship.

Literature on informal mentoring of college students scaffolds its advantage in promoting student success.^6^ Nevertheless, structured mentoring programs in educational institutions promote specific skills, attitudinal change, and instill research interest supporting professional development.^7^ Factors associated with effective mentoring programs in educational institutions include; visible goals and administrative support, continuous short and long-term evaluation, mentoring framework, regular meetings, mentor-mentee matching, ongoing mentorship training to elaborate program goals and expectations for building relationships.^8^ The faculty mentorship program thus designed to consider the rights of mentees, responsibilities of mentors, and human process was endorsed by both mentor and mentees.

Mentors of the program were enthusiastic to help the mentee’s in their professional development, offered support to set career goals and proposed transfer of knowledge, skills, and experiences to achieve goals, Results are supported by a study in which mentors valued their role in education and development of students.^9^ Mentors of the program improved their communication skills, leadership capabilities, and work performance supported by the role of mentors by Coyne-Foresi M et al.^10^ Literature also supports the training of mentors to further improve outcomes for optimally-mentored mentees.^11^

In mentoring models the mentors are expected to provide socio-emotional support for cognitive growth and personality development of mentees.^12^ However, this is a bidirectional process that benefits the professional and personal development of both mentors and mentees principally in the arena of health care.^13^ Both the mentors and the mentees in our study were aware of this fact; the mentees mentioned this in their expectations of the program and the mentors demonstrated their enthusiasm and help to set career goals for mentees, proposed transfer of knowledge, skills, and experience, and intended to offer new ideas and thinking. Research states that the mentee undeniably gets the benefit of learning under the influence, guidance, or direction given by the mentor. This holds even for diversified or minority students.^14^ Studies have confirmed that mentoring relationships are more helpful for mentees from diverse backgrounds with limited resources.^15^

Evaluation of mentoring programs has indicated positive career outcomes, and academic success.^12^,^16^ The mentors in our study also verbalized to play the same role in the professional and personal development of mentees. In a program similar to our study, experiences of mentors and mentees in a countrywide pharmacists program of the United Kingdom expressed satisfaction on the observed impact of the program on their personal as well as professional development and improvement.^17^ Other than professional development mentoring has been linked with positive interpersonal relationships and motivational outcomes.^10^ Evidence shows that the guidance and supervision of the mentor influence constructive attitudinal outcomes resulting in a positive attitude of the mentee towards the activity with positive behavioral outcomes.^18^,^19^

It has been observed that the assumed supervisory role adopted by the mentors gives them fulfillment at work through self-determination and self-reflection. Workplace mentoring contributes to familiarization with work ethics and culture,^20^ and relaxation of stress has been associated with psychological well-being and improved mental health.^21^ All these findings are in line with our study results where the mentors are willing to be role models for a positive behavior change in mentees but also impart meaning at work. This cordial relationship can therefore add to personal and professional development as well as emotional well being of mentees as has been recognized in a national study^22^.

### Strengths of the study

This study is unique which explores perception of mentors and mentees, challenges and way forward to improve upon the program,. These reflections may suggest strategies to ‘enhance’ comprehensive mentoring model in the Department of Biological & Biomedical Sciences, Aga Khan University and other universities

### Limitations of the study

This is preliminary information of the data, however, the effectiveness of mentoring program should include a systematic collection and analysis of the design, process, implementation, and outcomes.^1^,^22^

## CONCLUSION

Both mentors and mentees recognized the importance of the faculty mentorship program at AKU-MC for professional development and improved work performance. In-depth exploration of mentors’ and mentees’ perspectives and continuous support can be used to identify challenges and deficits in the structure of the program to devise appropriate strategies.

### Authors’ Contribution:

**RR** designed the study.

**FK** executed the project and she is also the accountable for the accuracy and integrity of the study.

**TSA** took part in design of methodology.

All authors read the manuscript and revised the content.
